# Maximal muscle strength and body composition are associated with bone mineral density in chinese adult males

**DOI:** 10.1097/MD.0000000000019050

**Published:** 2020-02-07

**Authors:** Fei Chen, Qi Su, Yulan Tu, Jun Zhang, Xinji Chen, Tingxiao Zhao, Yazeng Huang, Guokang Xu

**Affiliations:** a Department of Orthopedics, Fuyang First People's Hospital; b Department of Orthopedics, Zhejiang Provincial People's Hospital; c Hangzhou Medical College People's Hospital, Hangzhou; d School of Clinical Medicine, Wenzhou Medical College, Wenzhou, Zhejiang; e Bengbu Medical College, Bengbu, Anhui, China.

**Keywords:** body composition, bone mineral density, Chinese male adults, maximal muscle strength

## Abstract

The relationship between maximal muscle strength (MMS) and bone mineral density (BMD) in males remains unclear. Therefore, the aim of this cross-sectional study was to investigate the association of MMS, using 3 fundamental compound exercises, and body composition with BMD in Chinese male adults. One hundred forty-seven Chinese male adults aged 20 to 47 years were recruited. Total and regional BMD and body composition were measured using dual-energy X-ray absorptiometry. Measurements of MMS included bench press, deadlift, and squat 1-repetition maximum (1RM). Bench press, deadlift, squat 1RM, fat mass (FM), and lean mass (LM) had a significant positive association with BMD. Intriguingly, squat 1RM was found to have a stronger association than bench press or deadlift 1RM, whereas bench press 1RM was found as the strongest determinant of BMD at the forearm sites. Furthermore, LM was found to be stronger related with BMD than FM. Our findings identify LM, FM and MMS are positively associated with BMD and squat may serve as a simple, most efficient strategy to optimize peak total body BMD, while bench press fit best for forearm BMD. Our results validate the benefits of MMS training in males and underscores site-specific effects on BMD levels. These findings emphasize the need for prospective studies to investigate the maximum therapeutic potential and sex specific modifiers of MMS training.

## 1 Introduction

Osteoporosis is a disease characterized by reduced bone mass and microarchitectural defects whose cumulative effects result in brittle bones with an increased risk of fragility fractures.^[1]^ Approximately 9 million adults in the U.S. have osteoporosis and an additional 48 million have low bone mass, which underscores the public health importance of mitigating fragility fractures.^[2]^ Although osteoporosis disproportionately affects women, 1 out of 8 men over the age of 50 experience a fragility fracture, with the most common sites as forearm, spine, and hip.^[3,4]^ In contrast to the rapid bone loss experienced by postmenopausal women, men will undergo slower bone loss starting at the sixth decade of age at an average rate of 0.5% to 1.0% of total bone mass per year.^[5–7]^ Recently, the rising incidence of osteoporotic fractures in men has kindled interest in preventative measures for the male population.^[8]^

Current guidelines recommend strength training and weight-bearing exercises for the prevention of bone loss and maintaining bone mass in patients with osteoporosis.^[9]^ Both plyometric high-impact exercises and traditional strength training have been reported to increase bone mineral density (BMD) at the hip and lumbar spine in young adults.^[10–12]^ Resistance training has been suggested as an effective way for preventing bone loss.^[13–15]^ Maximal muscle strength (MMS), measured as 1-repetition maximum (1RM), has been reported to correlate with BMD in women.^[16–18]^ Maximal strength training has also been reported as a simple and effective training method for improvement of BMD in young adult women and postmenopausal women with osteoporosis or osteopenia.^[19,20]^ Furthermore, hand grip strength and leg power has been associated with increasing BMD at the lumbar spine and total hip in men, respectively.^[21]^ With accumulating evidence suggesting the association between muscle strength and BMD, which intervention is most effective remains unclear.

In this present study, we aimed to investigate the association between MMS and body composition and BMD in Chinese male adults. We aimed to find out association between MMS and site specific BMD.

## 2 Materials and methods

### 2.1 Participants

From January 2013 to May 2015, a total of 147 Chinese male adults were recruited in Zhejiang Provincial People's Hospital. All participants were from 20 to 47 years old. Those with known metabolic bone diseases or those under any medications likely to influence BMD were excluded from the study. Three candidates were excluded because of history of injury with their upper or lower legs. All of the participants have at least 1-year experience with strength training and are familiar with exercises such as the bench press, deadlift, and squat. All of the participants were informed about the details of procedure and written informed consent in accordance with the Declaration of Helsinki was obtained from all of the participants for the subsequent submission for publication. Our study was approved by the Ethics Committee of the Fuyang First People's Hospital and Zhejiang Provincial People's Hospital.

### 2.2 Measurements

All participants completed a questionnaire on demographic study. Participants who smoked at least 1 cigarette per day or drank alcohol once a week for at least 6 months were defined as smokers or drinkers. None of the participants were heavy smokers or drinkers. Physical measurements were obtained based on standardized protocol as previous described.^[22,23]^ Briefly, height was measured without shoes to the nearest 0.1 cm, weight with only light clothing to the nearest 0.1 kg (Detecto). All values were recorded as the mean of the 3 measures. BMI was calculated as body weight (in kilograms) divided by height (in meters) squared. Dual-energy X-ray absorptiometry ( software version 13.60.033; GE-lunar iDXA, WI) was used to measure lean mass (LM), fat mass (FM) and total and regional BMD through whole-body scans. Regional BMD refers to the mean BMD in the regions of forearm, spine and hip. DXA was calibrated daily using a standard phantom provided by the manufacturer.

MSS for bench presses, deadlifts and squats were determined for each participant by measuring the 1 RM for each exercise. Following multiple warm-up trials described previously,^[24]^ loading for the tests were increased or decreased by 2.5 to 5 kg based on subject's self-reported 1 RM performance during the previous 6 months. The rest periods between the trials were around 3 to 5 minutes to avoid possible fatigue. When a participant successfully performed a 1RM, weight was increased by 2.5 kg and the exercise was performed again until the participant was unable to perform the exercise. The traditional bench press was performed in the standard supine position on the flat bench (Impulse Fitness IT 7014, Shandong, China). Participants lowered the bar until touching the sternum and pressed the weight away from the torso until the elbows were fully extended. No pause between the descent and ascent of the barbell was required, and bouncing the weight off the chest disqualified that attempt. An acceptable deadlift was determined by the participants lifting the loaded barbell from the floor until their knees were fully extended. An acceptable squat was judged by lifting the weight through a range of motion in which the greater trochanter of the femur was lowered to the same level as the knee (quadriceps parallel to the floor) and coming back to a standing position in which, knees were fully extended. Two experienced spotters stood beside the barbell for safety during the whole exercise and verbally guided the participants to maintain proper form during the maximal lift. The heaviest weight for which a participant could successful perform a 1RM was recorded as the MMS for that exercise. We measured the 3 MMS in order of bench press, deadlift, and squat. The rest period between 2 different MMS measurements was 30 minutes to allow for adequate recovery. All the measurements of one participant were recorded in a single day and the results were collected by the same investigator.

### 2.3 Statistical analysis

Summary statistics of participants were presented as mean ± standard deviation or numbers (percentage, %) (Table 1) as appropriate. Pearson correlation analysis was used for testing the relationships between the predictors and regional and total BMD (Table 2). The associations between MMS and body composition with regional and total BMD (dependent variables) were tested by multiple linear regression, adjusting for age, height, weight, BMI, alcohol consumption and cigarette smoker (Table 3). Sample size calculation with an expected power of 0.95 and α- error of 0.05 was implemented for simple correlation and linear multivariate regression. For simple correlation, the null value and the minimal difference of the correlation were set to be 0 and 0.3, respectively, under the Fisher *Z* test. For linear multivariate regression, we had 7 predictors, in which one was of interest and would be tested, assuming a partial correlation of 0.4. One hundred thirty-eight samples are required for a simple correlation and 83 samples are required for linear multivariate regression, respectively. SPSS (version 16.0 for Windows, SPSS Inc., Chicago, IL) was used for analysis. All statistical tests were 2-tailed, and *P* < .05 were considered significant.

**Table 1:**
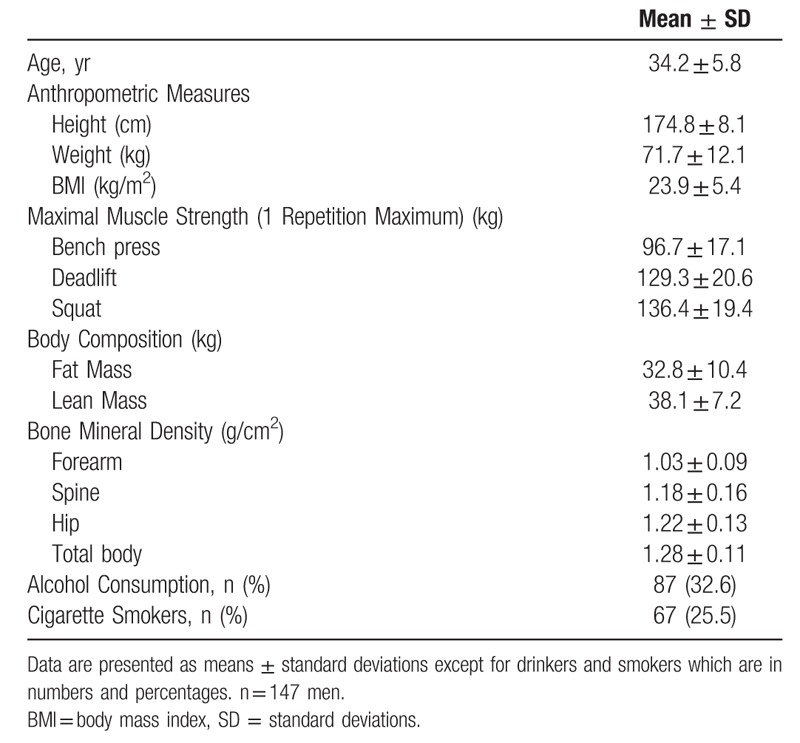
Characteristics of the subjects.

**Table 2:**
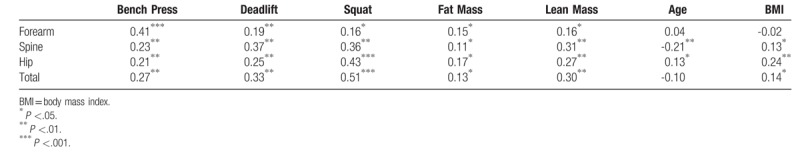
Correlations between maximal muscle strength, body composition and bone mineral density.

**Table 3:**
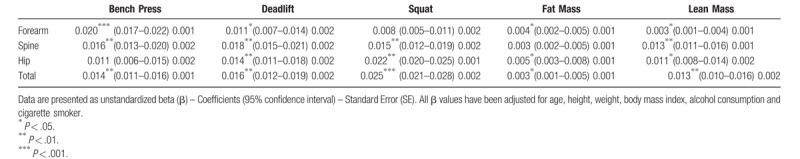
Association of maximal muscle strength, body composition and bone mineral density.

## 3 Results

### 3.1 Participant basic characteristics

The basic characteristics of 147 participants are shown in Table 1. All the participants were aged from 20 to 47 with an average of 34.2 ± 5.8 years. The height and weight of all participants were 174.8 ± 8.1 cm and 71.7 ± 12.1 kg, respectively. Percentages of alcohol drinkers and cigarette smokers were 32.6 and 25.5, respectively. Their MMS, body composition, regional and total BMD results are compiled in Table 1.

### 3.2 MMS and body composition are positively associated with BMD in Chinese male adults

Correlations between MMS, body composition and BMD are shown in Table 2. The 1RM of bench presses, deadlifts and squats were positively associated with forearm, spine, hip, and total BMD. Among them, squats have a stronger association with hip and total BMD than either the bench press or deadlift (*r* = 0.43, *P* < .001 versus *r* = 0.21, *P* < .01 and *r* = 0.25, *P* < .01 for hip BMD, while *r* = 0.51, *P* < .001 versus r = 0.27, *P* < .01 and *r* = 0.33, *P* < .01 for total BMD). The bench press has a stronger association with forearm BMD than the deadlift or squat (*r* = 0.41, *P* < .001 versus *r* = 0.19, *P* < .01 and *r* = 0.16, *P* < .01, respectively). Regarding body composition, LM has a stronger association with spine, hip and total BMD than FM (*r* = 0.31, *P* < .01 versus *r* = 0.11, *P* < .05, *r* = 0.27, *P* < .01 versus *r* = 0.17, *P* < .05 and *r* = 0.30, *P* < .01 versus *r* = 0.13, *P* < .05, respectively). Age is associated with spine and hip BMD (*r* = -0.21, *P* < .01 and *r* = 0.13, *P* < .05, respectively) while not with forearm and total BMD. BMI was associated with spine, hip and total BMD (*r* = 0.13, *P* < .05, *r* = 0.24, *P* < .01 and *r* = 0.14, *P* < .05, respectively) while not with forearm BMD, as shown in Table 2.

### 3.3 Controlling for MMS confounders maintains positive association with BMD

In order to mitigate the effects of potential confounders such as age, height, weight, BMI, alcohol consumption and cigarette smoking, multiple linear regression was performed including these as explanatory variables along with MMS, and body composition. Table 3 compiles the beta weights of the model. After adjusting for potential confounders, almost all of the MMS and body composition were positively associated with regional and total BMD, details shown as Table 3.

## 4 Discussion

In this study, we demonstrated that both MMS and body composition are significantly positively associated with regional and total BMD. Squat 1RM had a stronger association than bench press or deadlift with femoral neck BMD, whereas bench press 1RM had the strongest association with BMD at the forearm sites. Furthermore, LM body composition was more strongly associated with BMD than FM body composition.

Accumulating evidences demonstrates that muscle strength is positively related with BMD.^[16–18,25–27]^ Maximal strength training is therefore recommended as a simple and effective training method for improvement of BMD.^[19,20]^ However, a previous study demonstrates that squats and deadlifts are effective in increasing BMD in young healthy men, while similar benefits were not observed in women who followed the same protocol.^[15]^ This suggests that either the mechanism of the bone anabolic effects of MMS strength training are different among men and women or that the thresholds for reacting to MMS stimulus are different among the sexes. Additionally, MMS are correlated with regional BMD and not total BMD, implying local rather than systemic effects. Furthermore, while most studies were based on Caucasian populations and focused on women (especially on postmenopausal women), these results can not necessarily be extrapolated to Asian populations or men. Intriguingly, a study based on Chinese adolescent girls indicated that LM was a stronger independent determinant than FM of bone mineral content, whereas handgrip muscle strength was found to be a significant determinant of bone mineral content at the forearm.^[28]^ These observations are partially consistent with our findings in this study. We investigated the 1RM of the 3 most popular exercises used in strength training. MMS of bench press predominantly affects upper body strength, whereas deadlifts and squats targets core musculature and lower body strength. Our findings indicated that MMS of bench press, deadlift and squat were all positively associated with regional and total BMD. 1RM of squat was stronger associated with BMD than bench press or deadlift, whereas 1RM of bench press as the strongest determinant of BMD at the forearm sites. We also found both LM and FM were positively associated with regional and total BMD, whereas LM has stronger association with BMD than FM.

The major strength of our study is that all the measurements were obtained by DXA by 1 technician using 1 densitometer and all MMS were performed in the same gym by 1 fitness trainer. Additionally, no participant had taken a drug known to interfere with muscle strength, bone homeostasis, fat or lean tissue metabolism. Finally, all the participants were Chinese adult male population living in the same region. A limitation of this cross-sectional study is that causal inferences between MMS and BMD are not possible. Additionally, this study design cannot interrogate what magnitude of changes in MMS are necessary for therapeutic benefit. Furthermore, we did not analyze the lifestyle of participants, such as diet style, physical exercise, calcium supplementation, or vitamin D levels, which may also affect BMD. Additionally, our participants are all Chinese male adult and our results may not be generalized to other ethnicities. Finally, bone turnover markers were not measured in this study. Thus, while the association with increased BMD is purportedly due to the anabolic effects of strength training, suppression of bone catabolism cannot be strictly excluded.

To the best of our knowledge, this is the first investigation addressing the association between MMS and body composition with BMD in Chinese male adults. We conclude that MMS was positively associated with BMD. Our findings lend further support for high-load resistance training for increasing peak bone mass prior to age-related bone loss. Our results indicate that squats may serve as a simple exercise to incorporate in a strength training regimen to optimize peak total body BMD, while focusing on the bench press should be considered to mitigate forearm BMD. Further research should focus on longitudinal studies to determine if MMS training prior to the onset of bone mass decline can increase peak bone mass to levels that delay the onset of osteoporosis. Furthermore, additional research is needed to determine if beginning MMS straining after the onset of bone mass decline can rescue or slow the progression towards fragile bones. Finally, given that BMD response to MMS may be sex dependent, mechanistic studies focusing on the link between MMS and BMD in men and women are needed to fully elucidate causal relationships.

## Acknowledgments

I thank Dr. Michael-John G. Beltejar from University of Rochester Medical Center for his assistance and thoughtful recommendations. I thank Dr. Jiayin Zheng from Fred Hutchinson Cancer Research Center for his help with statistical analysis.

## Author contributions

**Conceptualization:** Fei Chen, Qi Su, Yulan Tu, Jun Zhang, Guokang Xu.

**Data curation:** Fei Chen, Qi Su, Yulan Tu, Tingxiao Zhao, Yazeng Huang.

**Funding acquisition:** Guokang Xu.

**Investigation:** Qi Su, Yulan Tu, Xinji Chen, Yazeng Huang, Guokang Xu.

**Methodology:** Fei Chen, Qi Su, Yulan Tu, Jun Zhang, Xinji Chen, Yazeng Huang, Guokang Xu.

**Writing – original draft:** Fei Chen, Qi Su, Yulan Tu, Jun Zhang, Guokang Xu.

**Writing – review & editing:** Xinji Chen, Tingxiao Zhao, Yazeng Huang, Guokang Xu.

## References

[1] NIH consensus development panel on osteoporosis prevention, diagnosis, and therapy, March 7-29, 2000: highlights of the conference. South Med J 2001;94:569–73. 11440324

[2] Nakamura T Takano T Fukunaga M Eldecalcitol is more effective for the prevention of osteoporotic fractures than alfacalcidol. J Bone Miner Metab 2013;31:417–22. 23575909 10.1007/s00774-012-0418-5 PMC3709079

[3] van Staa TP Dennison EM Leufkens HG Epidemiology of fractures in England and Wales. Bone 2001;29:517–22. 11728921 10.1016/s8756-3282(01)00614-7

[4] Schuit SC van der Klift M Weel AE Fracture incidence and association with bone mineral density in elderly men and women: the Rotterdam Study. Bone 2004;34:195–202. 14751578 10.1016/j.bone.2003.10.001

[5] Seeman E Clinical review 137: sexual dimorphism in skeletal size, density, and strength. J Clin Endocrinol Metab 2001;86:4576–84. 11600506 10.1210/jcem.86.10.7960

[6] Melton LJ 3rd Khosla S Achenbach SJ Effects of body size and skeletal site on the estimated prevalence of osteoporosis in women and men. Osteoporos Int 2000;11:977–83. 11193251 10.1007/s001980070037

[7] D’Amelio P Isaia GC Male osteoporosis in the elderly. Int J Endocrinol 2015;2015:907689. 26457082 10.1155/2015/907689 PMC4592737

[8] Madeo B Zirilli L Caffagni G The osteoporotic male: overlooked and undermanaged? Clin Interv Aging 2007;2:305–12. 18044181 PMC2685264

[9] Kohrt WM Bloomfield SA Little KD American college of sports medicine position stand: physical activity and bone health. Med Sci Sports Exerc 2004;36:1985–96. 15514517 10.1249/01.mss.0000142662.21767.58

[10] Kohrt WM Barry DW Schwartz RS Muscle forces or gravity: what predominates mechanical loading on bone? Med Sci Sports Exerc 2009;41:2050–5. 19812511 10.1249/MSS.0b013e3181a8c717 PMC3037021

[11] Martyn-St James M Carroll S Progressive high-intensity resistance training and bone mineral density changes among premenopausal women: evidence of discordant site-specific skeletal effects. Sports Med 2006;36:683–704. 16869710 10.2165/00007256-200636080-00005

[12] Martyn-St James M Carroll S Effects of different impact exercise modalities on bone mineral density in premenopausal women: a meta-analysis. J Bone Miner Metab 2010;28:251–67. 20013013 10.1007/s00774-009-0139-6

[13] Arce-Esquivel AA Ballard JE Effects of resistance training on bone and muscle mass in older women: a review. Sport Exerc Med Open J 2015;1:89–96.

[14] Maddalozzo GF Widrick JJ Cardinal BJ The effects of hormone replacement therapy and resistance training on spine bone mineral density in early postmenopausal women. Bone 2007;40:1244–51. 17291843 10.1016/j.bone.2006.12.059

[15] Almstedt HC Canepa JA Ramirez D Changes in bone mineral density in response to 24 weeks of resistance training in college-age men and women. J Strength Cond Res 2011;25:1098–103. 20647940 10.1519/JSC.0b013e3181d09e9d

[16] Cussler EC Lohman TG Going SB Weight lifted in strength training predicts bone change in postmenopausal women. Med Sci Sports Exerc 2003;35:10–7. 12544629 10.1097/00005768-200301000-00003

[17] Kerr D Morton A Dick I Exercise effects on bone mass in postmenopausal women are site-specific and load-dependent. J Bone Miner Res 1996;11:218–25. 8822346 10.1002/jbmr.5650110211

[18] Pasco JA Holloway KL Brennan-Olsen SL Muscle strength and areal bone mineral density at the hip in women: a cross-sectional study. BMC Musculoskelet Disord 2015;16:124. 26003407 10.1186/s12891-015-0586-2 PMC4493811

[19] Mosti MP Carlsen T Aas E Maximal strength training improves bone mineral density and neuromuscular performance in young adult women. J Strength Cond Res 2014;28:2935–45. 24736773 10.1519/JSC.0000000000000493

[20] Mosti MP Kaehler N Stunes AK Maximal strength training in postmenopausal women with osteoporosis or osteopenia. J Strength Cond Res 2013;27:2879–86. 23287836 10.1519/JSC.0b013e318280d4e2

[21] Schwarz P Jørgensen N Nielsen B Muscle strength, power and cardiorespiratory fitness are associated with bone mineral density in men aged 31-60 years. Scand J Public Health 2014;42:773–9. 25269791 10.1177/1403494814552119

[22] Zhang J Jin Y Xu S Associations of fat mass and fat distribution with bone mineral density in Chinese obese population. J Clin Densitom 2015;18:44–9. 24815308 10.1016/j.jocd.2014.03.001

[23] Shao H Xu S Zhang J Association between duration of playing video games and bone mineral density in Chinese adolescents. J Clin Densitom 2015;18:198–202. 25937308 10.1016/j.jocd.2015.02.007

[24] Wilson GJ Newton RU Murphy AJ The optimal training load for the development of dynamic athletic performance. Med Sci Sports Exerc 1993;25:1279–86. 8289617

[25] Marin RV Pedrosa MAC Moreira-Pfrimer LDF Association between lean mass and handgrip strength with bone mineral density in physically active postmenopausal women. J Clin Densitom 2010;13:96–101. 20171571 10.1016/j.jocd.2009.12.001

[26] Sherk VD Palmer IJ Bemben MG Relationships between body composition, muscular strength, and bone mineral density in estrogen-deficient postmenopausal women. J Clin Densitom 2009;12:292–8. 19155180 10.1016/j.jocd.2008.12.002

[27] Rhodes EC Martin AD Taunton JE Effects of one year of resistance training on the relation between muscular strength and bone density in elderly women. Br J Sports Med 2000;34:18–22. 10690445 10.1136/bjsm.34.1.18 PMC1724140

[28] Foo LH Zhang Q Zhu K Influence of body composition, muscle strength, diet and physical activity on total body and forearm bone mass in Chinese adolescent girls. Br J Nutr 2007;98:1281–7. 17640423 10.1017/S0007114507787421

